# Dual targeting of the tumor and its associated vasculature using a single bispecific chimeric antigen receptor molecule

**DOI:** 10.1186/2051-1426-2-S3-P6

**Published:** 2014-11-06

**Authors:** Tiara Byrd, Kristen Fousek, Antonella Pignata, Amanda Wakefield, Zakaria Grada, Kevin Aviles-Padilla, Bradley S Fletcher, Meenakshi Hegde, Brad St Croix, Nabil Ahmed

**Affiliations:** 1 Baylor College of Medicine, Houston, TX, USA; 2 Brown University, Providence, RI, USA; 3 University of Florida, Gainesville, FL, USA; 4 National Cancer Institute, Frederick, MD, USA

## Background

We have previously demonstrated the efficacy of HER2 specific chimeric antigen receptor (CAR) T cells in animal models of human cancer. While HER2 CAR T cells induced tumor regression, tumors recurred in a subset of animals. Lytic co-targeting of the tumor endothelium could represent a strategy that enhances tumor control. Tumor endothelium marker 8 (TEM8) is a recently described tumor endothelium-restricted antigen conserved in both mice and humans that represents an attractive antigen for vascular targeting [1].

## Purpose

To test the advantage of co-targeting TEM8 in conjunction with HER2 using a T cell product expressing a novel TEM8/HER2 bispecific CAR molecule.

## Methods

We designed, *in silico*, a single CAR molecule with both TEM8 and HER2-specific exodomains joined together in tandem (thus termed TanCAR). A retroviral construct encoding a TEM8 specific single chain variable fragment (scFv) from the mAb SB5, a Glycine/Serine linker and a HER2 specific scFv (mAb FRP5) exodomain, followed by a CD28 transmembrane domain, and a CD28.CD3-zeta signaling endodomain was transduced CD3/CD28-activated T cells with RD114-pseudotyped retroviral particles to generate TEM8/HER2 bispecific TanCAR T cells. CAR expression was confirmed by flow cytometry. Standard immunoassays were used to test the CAR T cell functionality.

## Results

TEM8/HER2 TanCAR molecules were expressed on the surface of up to 90% of primary T cells. Staining specific for FRP5 and SB5 ensured the expression of the TanCAR molecule in its entirety. TanCAR T cells selectively recognized and killed TEM8 and HER2 positive targets distinctly, as evidenced by the release of the immuno-stimulatory cytokines interferon-gamma and interleukin-2 *in vitro *and standard 4 hour ^51^Cr release cytotoxicity assays. Cytokine release and killing were significantly enhanced when TanCAR T cells encountered both target antigens simultaneously. The kinetics of T cell activation followed a second order kinetic equation denoting a superadditive or synergistic effect upon recognition of a second antigen. Minimal activation or cytolytic activity occurred with target negative controls or with CAR null T cells.

## Conclusion

Co-targeting the tumor and its vasculature using bispecific TanCAR T cells could enhance activation of these cells and potentially be used to improve tumor control with therapeutic application in cancer patients.
